# Graph Cut-Based Human Body Segmentation in Color Images Using Skeleton Information from the Depth Sensor

**DOI:** 10.3390/s19020393

**Published:** 2019-01-18

**Authors:** Jonha Lee, Dong-Wook Kim, Chee Sun Won, Seung-Won Jung

**Affiliations:** 1 Department of Multimedia Engineering, Dongguk University, Pildong-ro 1gil 30, Jung-gu, Seoul 100-715, Korea; jonha.lee@samsung.com (J.L.); kimdongwook@dongguk.edu (D.-W.K.); 2 Department of Electronics and Electrical Engineering, Dongguk University, Pildong-ro 1gil 30, Jung-gu, Seoul 100-715, Korea; cswon@dongguk.edu

**Keywords:** depth image, graph cut, human body segmentation, image segmentation, Kinect sensor, skeleton

## Abstract

Segmentation of human bodies in images is useful for a variety of applications, including background substitution, human activity recognition, security, and video surveillance applications. However, human body segmentation has been a challenging problem, due to the complicated shape and motion of a non-rigid human body. Meanwhile, depth sensors with advanced pattern recognition algorithms provide human body skeletons in real time with reasonable accuracy. In this study, we propose an algorithm that projects the human body skeleton from a depth image to a color image, where the human body region is segmented in the color image by using the projected skeleton as a segmentation cue. Experimental results using the Kinect sensor demonstrate that the proposed method provides high quality segmentation results and outperforms the conventional methods.

## 1. Introduction

Segmentation of the human body regions is essential in several applications. For example, segmented human bodies can be synthesized with a scene of another environment for immersive virtual reality games and telepresence applications. Moreover, segmented human bodies can be useful for human activity recognition, enabling efficient security and video surveillance applications. However, due to the complicated shape and motion of human body parts, the automatic segmentation of a human body remains a challenging problem. Alternatively, a chroma key that uses a green screen or blue screen as a background has been widely-used [1]. The chroma key-based method makes human body segmentation straightforward, since the foreground region can be readily extracted by comparing the pixels with the predetermined background color. However, it is required to support an automatic human body segmentation under general background environments. 

Image segmentation is one of the most widely studied computer vision problems. In particular, there have been several research endeavors to apply image segmentation to a specific human body segmentation problem [2,3,4,5]. In Reference [2], a body pose is estimated from a color image and a human body region is segmented using the super-pixel segmentation and part appearance map. In Reference [3], face, torso, and limbs are detected to estimate the body pose and the graph-cut algorithm is used to extract the human body region. In Reference [4], a machine learning approach is used to extract the characteristics of the human shape. In Reference [5], human body skeletons are provided by user clicks and human body boundary lines are found using many anthropometrically estimated parameters. In cluttered background environments, however, these methods cannot accurately estimate the human body pose and thus the resultant segmentation can be unreliable. 

Depth sensors, such as Microsoft Kinect, have been very successful in the gaming industry. The depth sensors have also shown significant advantages in many applications, such as image rendering [6], image segmentation [7], object tracking [8], activity recognition [9], and image enhancement [10]. Especially for human body segmentation, the depth image can greatly facilitate the segmentation procedure. For example, the background subtraction technique can be applied to the depth image to filter out the pixels using the background depth values [11]. 

Since many of the aforementioned applications require a human body segmentation result of the color image, the segmentation result of the depth image cannot be directly used. Instead, the depth image can be used to help human body segmentation in the color image. In Reference [12], both color and depth images are used to obtain the initial body skeletons and human body segmentation with rough boundaries. The initial body skeletons are then refined using the body part labels and a kinematic model. The most closely related method to ours is an adaptive multi-cue fusion framework [13]. Both color and depth images are used to obtain the foreground region with precise boundaries, but the conventional multi-cue fusion framework tends to correct only mislabeled pixels around the initial foreground mask, and it is not tailored to the human body segmentation problem. 

In this paper, we present a dedicated solution for human body segmentation using a pair of color and depth images. In particular, we present an algorithm that uses the human body skeleton obtained from a depth image as a shape prior in the graph-based optimization. Experimental results show the effectiveness of the algorithm for human body segmentation.

The rest of the paper is organized as follows. Section 2 describes the conventional graph-based segmentation approach that we adopted. Section 3 presents the proposed method, Section 4 provides our experimental results, and Section 5 concludes the paper.

## 2. Graph Cut-Based Segmentation

Graph algorithms have been widely used for image segmentation problems [14,15,16,17]. By treating a pixel or super-pixel as a node and assigning an edge to neighboring pixels or super-pixels, a graph structure can be established from an image. Image segmentation is then performed by dividing nodes into multiple groups according to a certain energy minimization criterion. To this end, image segmentation is typically formulated as a pixel labeling problem, where different labels indicate different group identifiers. Let *L* denote the pixel label vector, which is defined as follows:

*L* = (*L*_1_, · · ·, *L_x_*, · · ·, *L_N_*), (1)

where *N* represents the number of pixels in the image and *L_x_* denotes a label of pixel *x* (i.e., *L_x_* = 0 for the background and *L_x_* ≠ 0 for the foreground). The energy function to be minimized is defined as follows:

(2) $$E\left(L\right)=\;\lambda \underset{x\in X}{\sum }E_{D}\left(L_{x}\right)\;+\underset{(x,y)\in \Omega }{\sum }E_{S}\left(x,y\right)\delta \left(L_{x},L_{y}\right),$$

where

(3) $$\delta \left(L_{x},L_{y}\right)=\;\{\begin{array}{c} 0,\;if\;L_{x}=L_{y},\\ 1,\;\;otherwise. \end{array}$$

In Equation (2), *X* is a set of all pixels, *Ω* is a neighborhood system, and *λ* is a scalar value that specifies the importance of the data term *E_D_* against the smoothness term *E_S_*. The data term accounts for the fitness of the label *L_x_* at pixel *x*, which is defined as follows:

(4) $$E_{D}\left(L_{x}\right)=\;-\ln p(I_{x}|L_{x}).$$

In other words, how the intensity or color at pixel *x*, *I_x_*, fits into the background/object histogram [14] or the Gaussian mixture model (GMM) [14] is measured as a negative log-likelihood. The smoothness term is used to enforce the same label for the neighboring pixels with similar colors, which is typically defined as follows:

(5) $$E_{S}(x,y)=\exp\left(-\frac{{\left\|I_{x}-I_{y}\right\|}^{2}}{2\sigma^{2}}\right),$$

where *σ* denotes a standard deviation value and $\left\|I_{x}-I_{y}\right\|$ measures the Euclidean distance between the two color vectors. The graph-based segmentation algorithm has been used extensively, especially with interactive segmentation, where the user-provided segmentation seeds are used in defining the data term. For example, the GrabCut segmentation [15] requires a bounding box of the object to determine the pixels to be used in GMMs.

**Figure 1:**
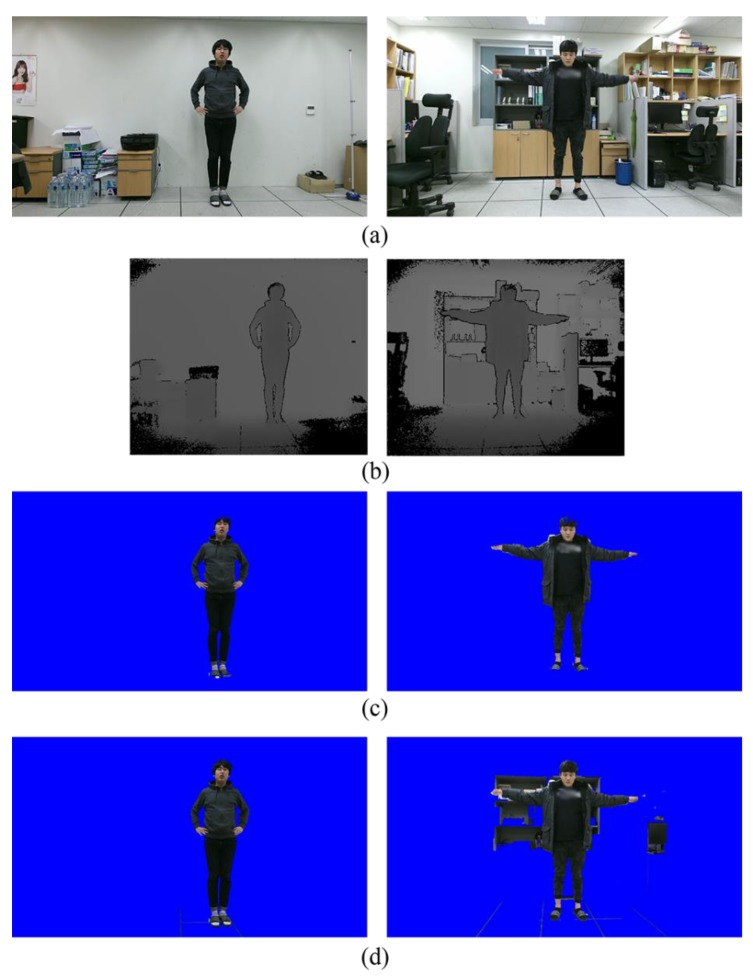
Result of the graph-based segmentation: (**a**) Color images (1920 × 1080), (**b**) depth images (512 × 424), (**c**) foreground regions, (**d**) segmentation results obtained by the graph-based segmentation algorithm [14]. Background regions are blue-colored.

In our application scenario, the aforementioned approach can be applied without explicit user interaction. Once initial human body regions are obtained from the depth image, we can first project them onto the color image and treat them as segmentation seeds. The segmentation result in the color image can then be obtained by minimizing Equation (2). Figure 1 shows the example obtained using the Microsoft Kinect v2 sensor (Microsoft Corporation, Redmond, WA, USA). The color and depth sensors of the Kinect are used to obtain color and depth image pairs, as shown in Figure 1a,b. Using the software development kit (SDK) of the Kinect, the foreground regions are then extracted from the depth image and then projected the foreground region to the color image, as shown in Figure 1c.

Due to the performance limitation of the depth sensor, the foreground region extracted from the depth image is not accurate enough. Under the simple background (first column in Figure 1), the graph-based segmentation method [14] provides a high quality segmentation result. However, under the cluttered background (second column in Figure 1), many background pixels that have similar colors with the foreground are mislabeled as the foreground, as shown in Figure 1d. Without any prior knowledge, this problem cannot be easily solved. 

## 3. Proposed Method

One effective way to improve the segmentation accuracy is to use a shape prior [18,19]. If the shape prior is included in the energy minimization framework, we can obtain the segmentation result that complies with the shape prior. In our application of human body segmentation, the skeleton data can be one of the best candidates for the shape prior and the skeleton information can be readily obtained in depth images. In particular, we use a Microsft Kinect v2 device with SDK, which has been extensively used in various applications [20,21,22]. The Kinect SDK extracts the foreground region for extracting the skeleton in real-time using the random forest classifier [11]. The skeleton in the depth image is then projected to the color image coordinate [23], and the projected skeleton is used as the shape prior.

**Figure 2:**
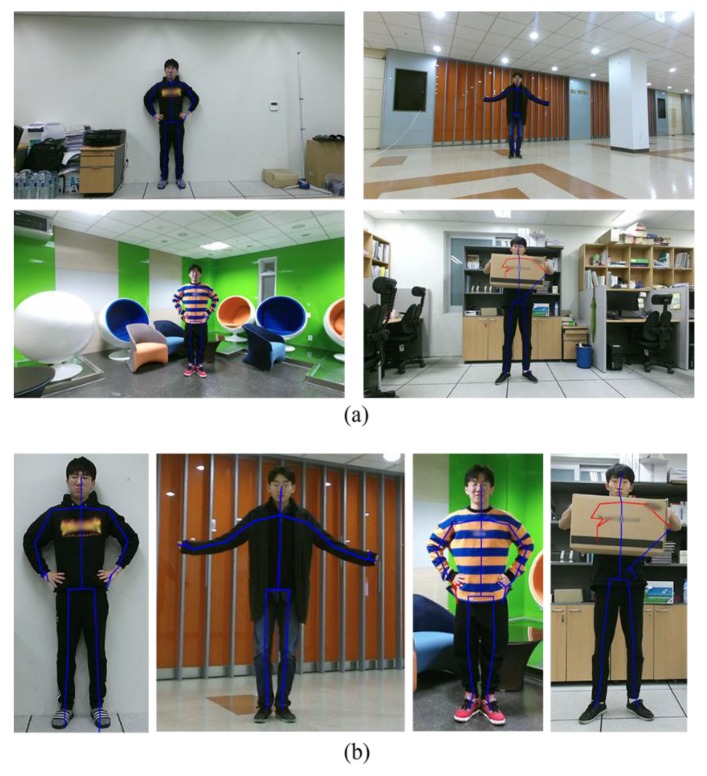
(**a**) Examples of the color images augmented with the skeleton and (**b**) their magnified sub-regions.

The remaining problem is how to model the shape prior. Figure 2 shows the estimated skeletons overlaid on the color images. Although the skeleton joint positions are not perfectly matched with the actual joint positions, the skeletons are included in the human body region. We thus modify the data term of Equation (4) in a way that the pixels close to the skeleton are labeled as the foreground. 

Let Φ*_l_* denote a binary map with the same size of the color image whose value is 1 at pixel *x* if *x* is on one of the lines connecting the joints of the *l*-th object. The Bayes rule leads us that *p* (*L_x_* = *l* | *I_p_*) ∝ *p* (*I_p_* | *L_x_* = *l*) *p* (*L_x_* = *l*). Here *p* (*L_x_* = *l*) is a prior probability of *x* being labeled as *l*, which is determined as follows:

(6) $$p(L_{x}=l)=\exp\left(-\frac{d^{2}(x,\Phi_{l})}{2\sigma_{J}^{2}}\right),$$

where *σ_J_* is the standard deviation and *d*(*x*, Φ*_l_*) represents the distance between *x* and Φ*_l_*. Specifically, the L1 distance between *x* and its closest pixel in Φ*_l_* whose value is 1 is computed by the distance transform [24]. The probability of *x* being labeled as *l* exponentially decreases as the distance from the skeleton of the *l*-th object increases. The probability of *x* being labeled as the background is defined as

(7) $$p(L_{x}=0)=1-\underset{l\in \{1,2,\cdot \cdot \cdot ,M\}}{\max}p(L_{x}=l),$$

where *M* represents the number of human bodies detected in the scene. The prior probabilities for each pixel *x* are normalized such that they all sum to 1.

In Equation (6), the parameter *σ_J_* determines the effect of the shape prior. In general, with higher reliability, a smaller value of *σ_J_* is desired. Since the reliability of each skeleton joint cannot be the same, we assign different *σ_J_* values to different joints. To this end, we use the Kinect SDK to classify the skeleton joints into the three states: Tracked, inferred, and non-tracked [11]. First, we make the non-tracked joints and the pixels connected to the non-tracked joints have the zero value in *Φ_l_* to prevent any abuse of the shape prior. Second, we adjust *σ_J_* values as *σ_J,t_* for the tracked state and as *σ_J,i_* for the inferred state, where *σ_J,i_* > *σ_J,t_*. For each pixel *x*, we find its closest joint and adjust *σ_J_* value according to the state of the closest joint. In Figure 2, the lines connected to the joints with the tracked-state and the inferred-state are blue-colored and red-colored, respectively.

In addition, the scattering of the emitted infrared light reduces the reliability of depth estimates in both time-of-flight (ToF) and structured light-based depth sensors. In particular, the reliability of the skeleton joints is especially low at the head and foot joints, due to the scattering problem. We thus set *σ_J_* as *σ_J,hf_* for the head and foot joints although when they are with the tracked state, where *σ_J,hf_* > *σ_J,t_*. Our parameter adjustment scheme is simple and heuristic, but essential, for the better use of the shape prior. We will compare the results obtained with/without varying *σ_J_* values in Section 4. 

Finally, the graph-based optimization is performed by minimizing the energy function in Equation (2) with the following modified data term.

(8) $$E_{D}(L_{x})=-\ln p(I_{x}|L_{x})-\ln p(L_{x}).$$

Owing to the modified data term, human body segmentation can perform robustly against the cluttered environments. Except for the data term, the other parts in Reference [14] remain unchanged to evaluate the effectiveness of our modification only. Note that the proposed method can be applied to any advanced graph optimization methods for further performance improvement.

For the human body segmentation of video sequences, we apply the proposed method to each frame individually. Although video segmentation can be performed using the three-dimensional (3-D) graph structure [25], such a method is computationally expensive and memory demanding. We found that the frame-by-frame segmentation is sufficient for human body segmentation owing to the robust shape prior provided by the depth image.

## 4. Experimental Results

**Figure 3:**
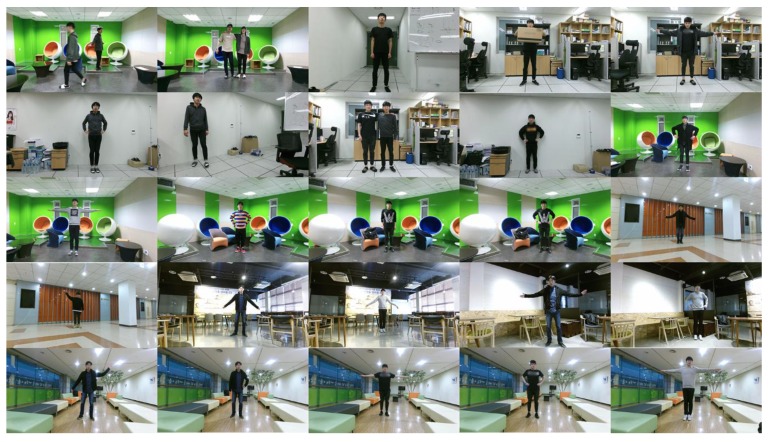
Test images of 25 difference scenes.

We constructed our own database that consists of 25 scenes, as shown in Figure 3. Each scene contains one or two persons with different body poses. The Microsoft Kinect v2 sensor was used to obtain the color and depth images and extract the skeletons [11]. The skeletons found from the depth images were projected to the color image coordinates using the method of [23]. For the performance evaluation, direct projection of the foreground region detected from the depth image to the color image (PROJ) [11,23], graph-based segmentation (GSEG) [14], GrabCut in one cut (OCUT) [16], and multi-cue fusion method (MFUS) [13] were compared with the proposed method. 

**Figure 4:**
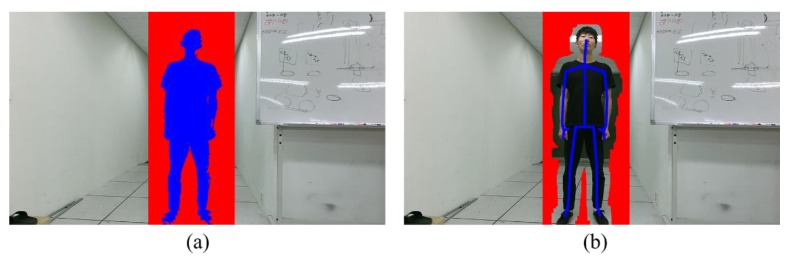
Foreground (blue) and background (red) seeds for (**a**) the graph segmentation method [14] and (**b**) one cut segmentation method [16].

For GSEG, the color model of each object was generated from the region of PROJ. The rectangular region whose height and width are 40% larger than those of the bounding box containing the region of PROJ was constructed. The pixels inside the rectangular region, but outside the region of PROJ, were used for the background color modeling [14], as shown in Figure 4a. Here the GMMs were used for object and background color modeling [15] and the default parameter settings were used for the energy optimization in Equation (2) [24]. The original MFUS was modified by projecting the initial segmentation map to the color image in order to apply the segmentation at the color image resolution. Note that MFUS used consecutive color and depth frames for the segmentation. The original OCUT requires user-provided segmentation seeds. We instead generated the foreground and background segmentation seeds without user inputs. To this end, the skeleton in the color image was expanded using the dilation operator with the 5 × 5 structural element. The expanded skeleton was used as the foreground segmentation seed. For the background seed construction, the rectangular region whose height and width are 40% larger than those of the bounding box containing the region of PROJ was constructed. The region of PROJ was expanded using the dilation operator with the 51 × 51 structural element, and the pixels inside the rectangular region, but outside the expanded region of PROJ, were used as the background seed, as shown in Figure 4b. In other words, we made necessary modifications to OCUT for the performance comparison, and all the experimental parameters were fine tuned to obtain the best segmentation accuracy. The proposed method was implemented in the same manner of GSEG with the modified data term in Equation (8). The parameters of the proposed method were empirically chosen as $\sigma_{J,t}^{2}$ = 10, $\sigma_{J,i}^{2}$ = 80, and $\sigma_{J,hf}^{2}$ = 50. 

For the quantitative performance evaluation, we measured the Jaccard coefficient [26], which is defined as follows:

(9) $$J(L_{gt},L_{est})=\frac{\left|L_{gt}\cap L_{est}\right|}{\left|L_{gt}\cup L_{est}\right|},$$

where *L_gt_* and *L_est_* denote the ground-truth and estimated binary segmentation maps, respectively. We manually generated the ground-truth binary maps for all scenes in Figure 3 using an image editing software. ∩ and ∪ denote the intersection and union operators, respectively, and |⋅| measures the number of ones in the segmentation map. Here the binary segmentation map treats different human body regions as the same foreground, and thus the Jaccard coefficient can be measured for the scenes with single or multiple persons. 

**Figure 5:**
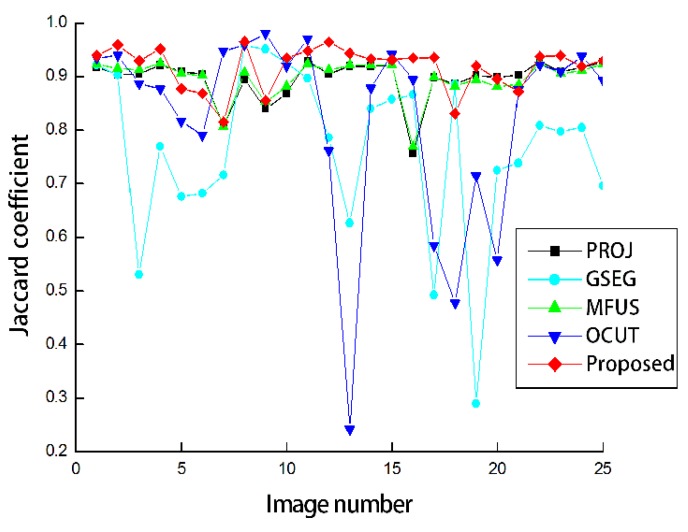
The Jaccard coefficients for the 25 scenes shown in Figure 3.

Figure 5 shows the Jaccard coefficients obtained by the five compared methods. The foreground regions obtained from the depth images were considerably accurate as can be seen from the result of PROJ. The average Jaccard coefficient was obtained as 0.896. GSEG applies the graph-cut segmentation to improve the segmentation accuracy, but the average Jaccard coefficient was even decreased by 0.130 compared to PROJ. This is because the segmentation failed for the scenes with complicated backgrounds. MFUS could only refine the mislabeled pixels around the initial object boundaries, and therefore the improvement was restricted. The average Jaccard coefficient was obtained as 0.897. Our application of OCUT to human body segmentation was not as asuccessful as the average Jaccard coefficient was obtained as 0.825. In the same manner of PROJ, OCUT also failed in the complicated scenes. Our modified data term could contribute to the significant performance improvement over PROJ as the obtained average Jaccard coefficient was the highest, at 0.917. When we set the standard deviation values to be the same as $\sigma_{J,t}^{2}$ = $\sigma_{J,i}^{2}$ = $\sigma_{J,hf}^{2}$ = 30 or 50, the average Jaccard coefficients were obtained as 0.880 or 0.836, respectively. In other words, it is necessary to adjust standard deviations according to the reliability of the skeleton joints. 

**Figure 6:**
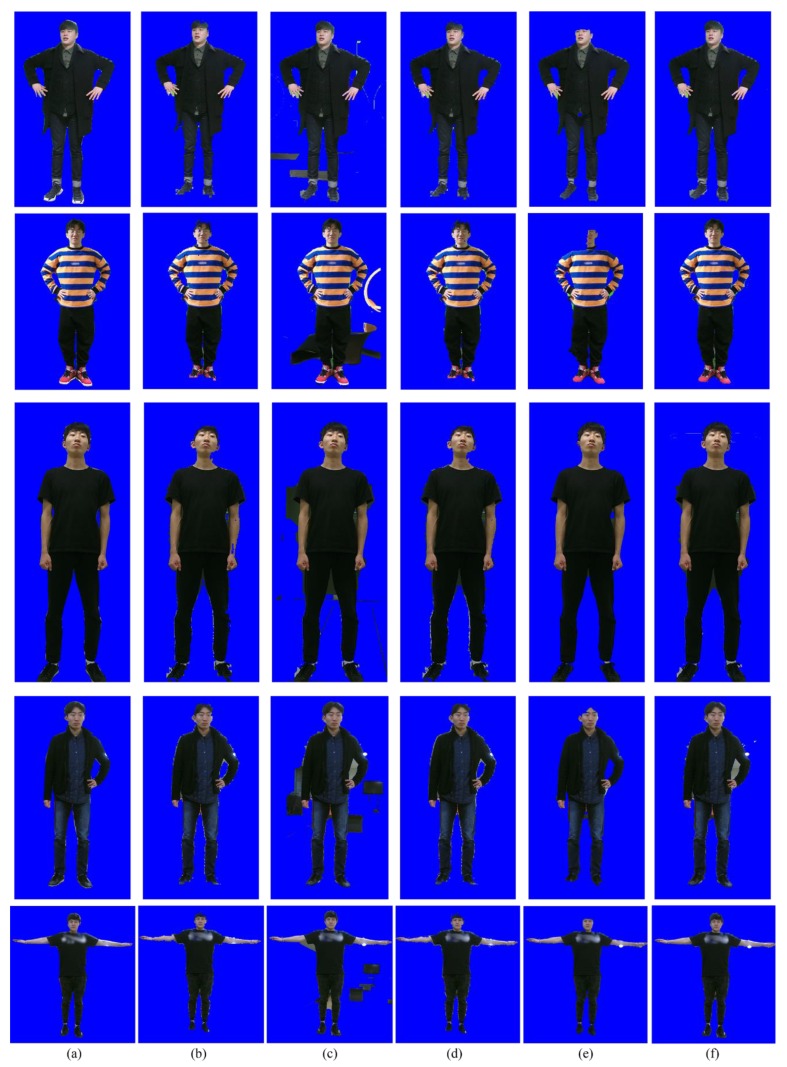
Comparison of the segmentation results: (**a**) Ground-truth, (**b**) PROJ [11], (**c**) graph-based segmentation (GSEG) [14], (**d**) multi-cue fusion method (MFUS) [13], (**e**) OCUT [16], and (**f**) the proposed method. The results are best viewed in the electronic version.

**Figure 7:**
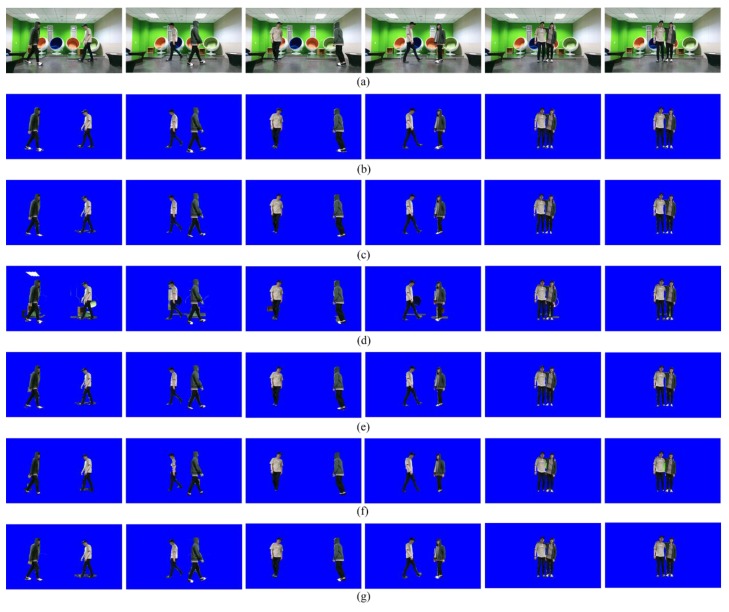
Results on image sequences: (**a**) Five sampled images out of 100 frames, (**b**) ground-truth segmentation maps, segmentation results obtained by (**c**) PROJ, (**d**) GSEG, (**e**) MFUS, (**f**) OCUT, and (**g**) the proposed method. The results are best viewed in the electronic version.

**Figure 8:**
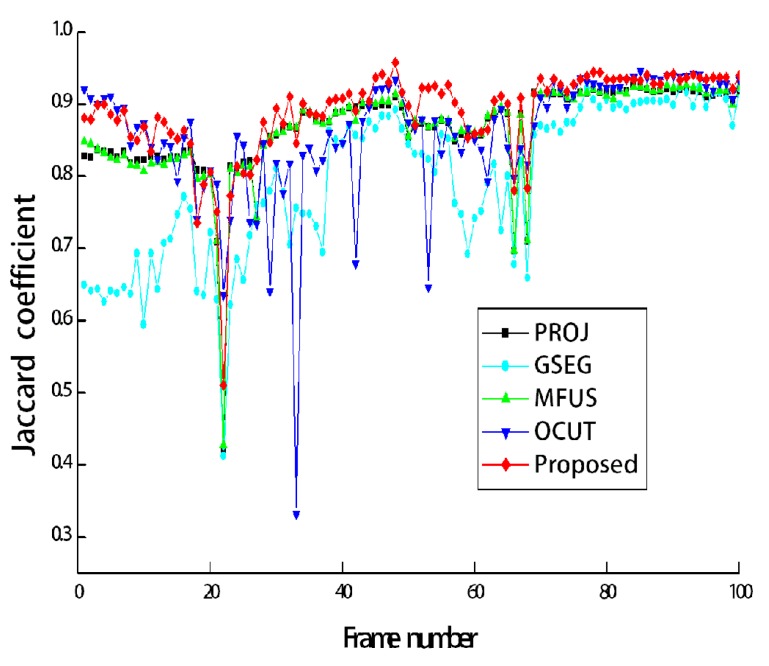
The Jaccard coefficients for the image sequences corresponding to Figure 7.

Figure 6 compares the segmentation results for the five methods. As can be seen from Figure 6b, the segmentation accuracy of PROJ is stable, but not accurate enough. Due to the performance limitation of the depth sensor, the foreground regions extracted from the depth image contain crude boundaries. GSEG tends to improve the segmentation accuracy, but it fails when the background and foreground share the similar color distribution, as shown in Figure 6c. MFUS can correct mislabeled foreground and background pixels, but it cannot recover a large amount of erroneous labels, as shown in Figure 6d. OCUT fails when the background and foreground share the similar color distribution or when the position of the skeleton is not accurate enough, as shown in Figure 6e. The proposed method yields the best segmentation accuracy, as shown in Figure 6f. Figure 7a shows the test image sequences that correspond to the first scene of Figure 3. The manually generated ground-truth maps are also shown in Figure 7b. During 100 frames, the two persons walked toward the other’s position, then passed by, and finally met together to simulate a challenging scenario. All the compared methods are single image-based, and thus they were applied to image sequences in a frame-by-frame manner. The segmentation accuracy can be compared from Figure 7c–g. Figure 8 shows Jaccard coefficients for 100 frames, which are obtained by comparing each segmentation result with its corresponding ground-truth map. The average Jaccard coefficient is obtained as 0.866, 0.794, 0.866, 0.861, and 0.889 for PROJ, GSEG, MFUS, OCUT, and the proposed method, respectively.

Last, we measured the processing time of different segmentation methods using a PC with an Intel Core™ i5-4670 3.40 GHz CPU (Intel Corporation, Santa Clara, CA, USA), and 16-G RAM (Samsung Electronics, Suwon, Korea). In our unoptimized implementation of GSEG, MFUS, OCUT, and the proposed method, PROJ was commonly used to obtain initial estimates. We thus first stored all the results of PROJ, which were mainly obtained using the SDK functions [11,23], and then applied the other methods as offline processing. For 100 consecutive frames used in Figure 8, the average processing time of segmenting one image was obtained as 2.59 s, 11.28 s, and 1.53 s for GSEG, OCUT, and the proposed method, respectively. Note that we included the shape prior to the energy function of GSEG, but the proposed method was found to be more computationally efficient than GSEG. This is because the proposed shape prior could make the graph-based optimization process stable and enabled convergence earlier than GSEG. The original MFUS showed the real-time performance of about 30 frames per second [13], but we modified the original MFUS to apply segmentation at the color image resolution. In our experiment, we mostly paid attention to the accurate reproduction of MFUS, and our implementation of MFUS required more than one minute for segmenting one image. The source code of the proposed method is provided as Supplementary Materials. 

## 5. Conclusions

An algorithm that uses the human body skeleton obtained by the depth image for the human body segmentation in a color image is presented. By defining the shape prior to the position of the human body skeleton and then applying the shape prior to the graph-based energy minimization framework, the human body region can be found around the human body skeleton. Experimental results demonstrated the superiority of the proposed method. Although our proposed method is based on the Kinect devices, it is straightforward to replace the SDK functions with our own methods to make the proposed method generally applicable. Moreover, recent deep learning-based skeleton estimation techniques using only color images show a promising result. As a future work, we also plan to extract skeletons and human body regions from only color images.

## Supplementary Materials

The supplementary materials are available online at http://www.mdpi.com/1424-8220/19/2/393/s1.
