# Supplementary figures and images for: Graph Cut-Based Human Body Segmentation in Color Images Using Skeleton Information from the Depth Sensor
Source: Sensors (Basel). 2019 Jan 18;19(2):393. doi: 10.3390/s19020393 (PMC6358916; doi:10.3390/s19020393)

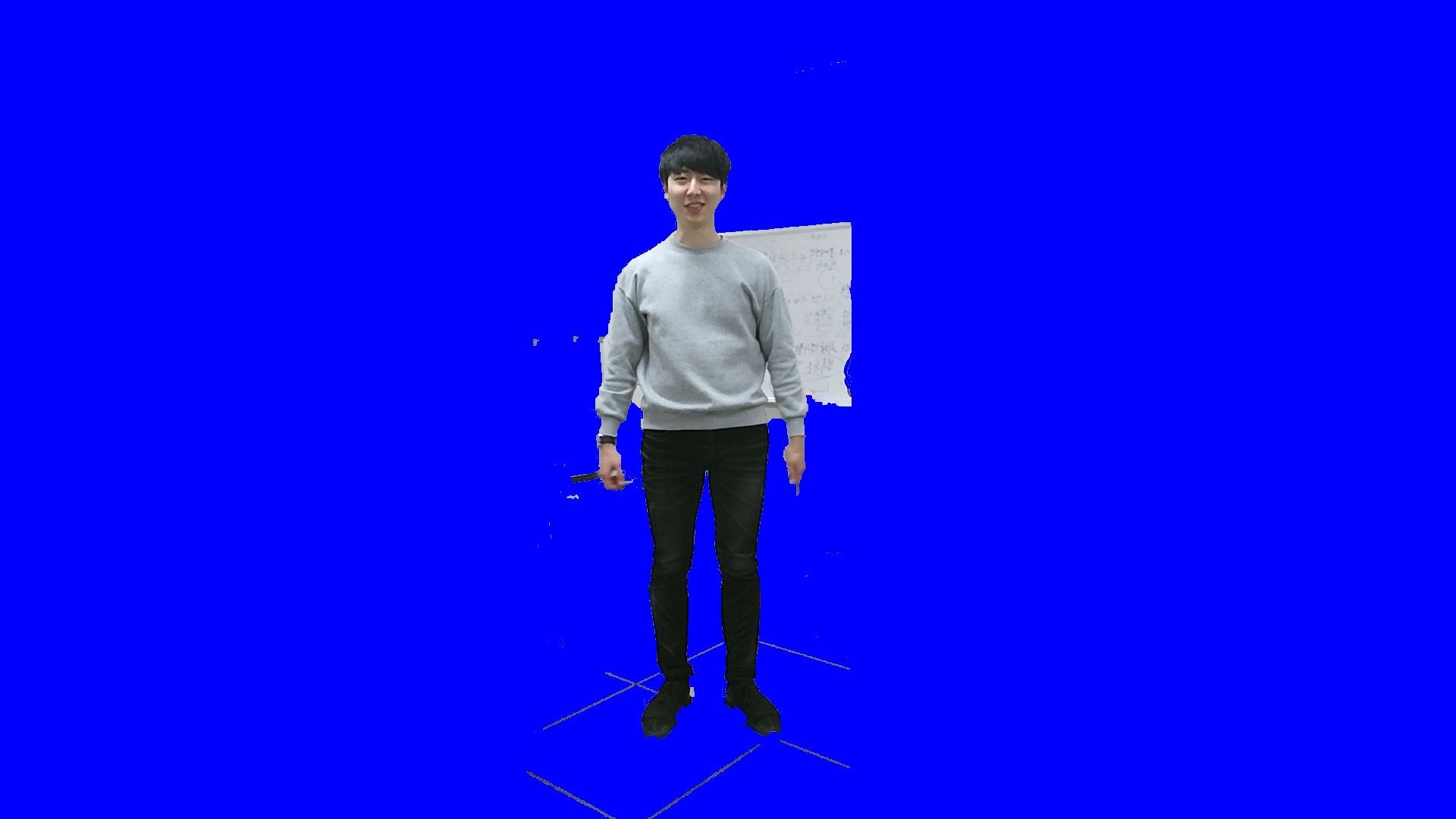

Supplement: Supplementary file 1 [file sensors-19-00393-s001.zip › Project/Project/Gseg_result.jpg]

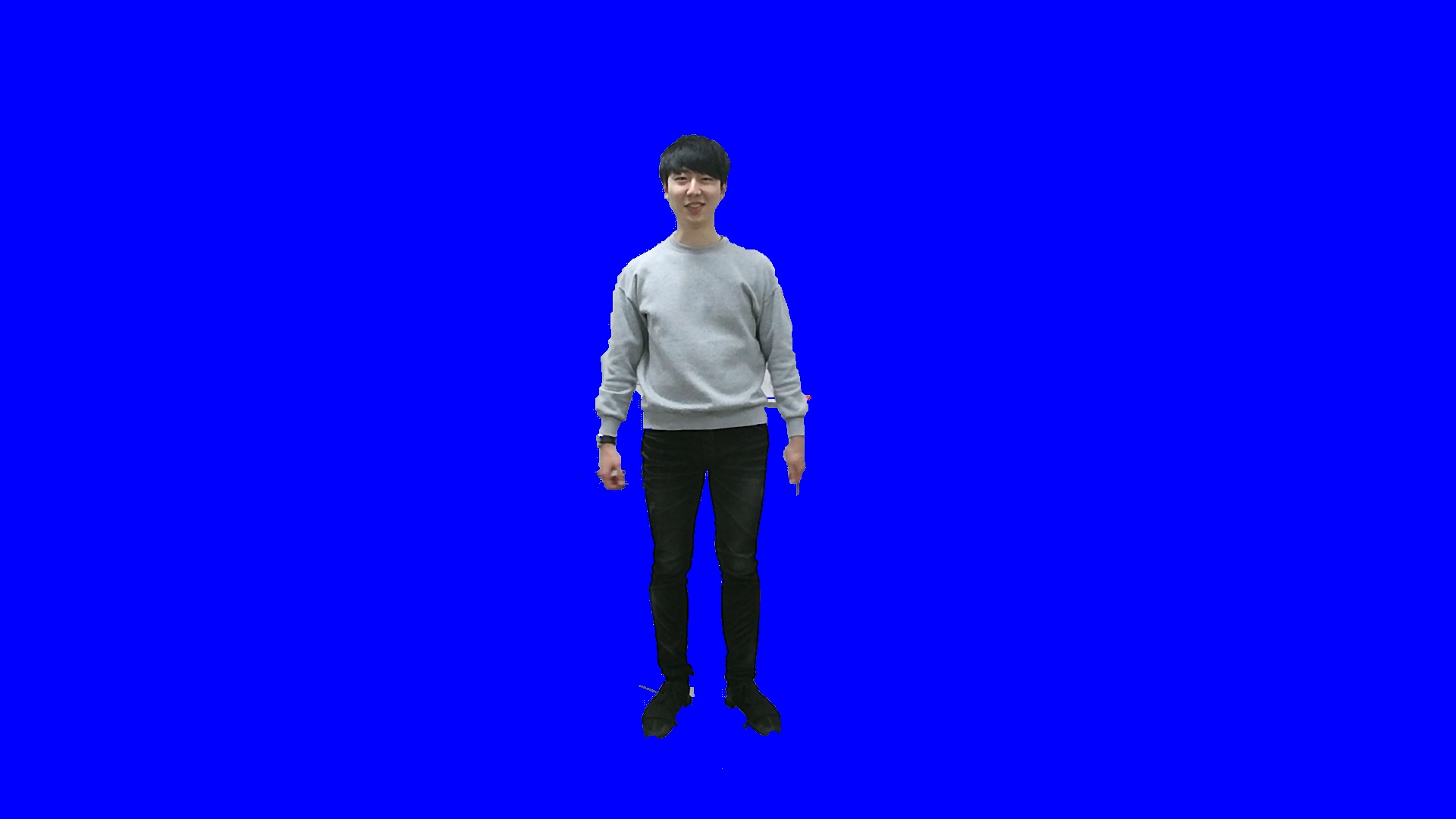

Supplement: Supplementary file 1 [file sensors-19-00393-s001.zip › Project/Project/prop_result.jpg]
